# Draft genome sequence and analysis of endophyte *Bacillus amyloliquefaciens* strain Can02R isolated from the roots of *Chenopodium album* L.

**DOI:** 10.1128/mra.01075-24

**Published:** 2024-12-17

**Authors:** Vladimir K. Chebotar, Anna V. Myskova, Margarita Khudyaeva, Oksana V. Keleinikova, Maria E. Baganova, Alexander N. Zaplatkin, Veronika N. Pishchik, Elena P. Chizhevskaya

**Affiliations:** 1 All-Russia Research Institute for Agricultural Microbiology, Pushkin, Petersburg, Russia; 2 Department of Genetics and Biotechnology Faculty of Biology, Saint Petersburg State University, Saint Petersburg, Russia; California State University San Marcos, San Marcos, California, USA

**Keywords:** whole genome, sequence, endophyte, *Bacillus amyloliquefaciens*, *Chenopodium album*

## Abstract

In this study, we sequence, assemble, and analyze the genome of endophyte *Bacillus amyloliquefaciens* Can02R isolated from the roots of the resurrection plant host, *Chenopodium album*. The assembly of the strain’s genome amounts to 3,965,760 bp and contains 3,989 coding sequences, among which synthetic antibiotic clusters and multidrug resistance transporters can be found.

## ANNOUNCEMENT

*Bacillus amyloliquefaciens* is a member of the *Bacillus subtilis* group, which includes the species of the Gram-positive spore-forming bacteria (phylum Firmicutes), many of which are associated with plants. They have a remarkable potential for the biosynthesis of adaptive molecules, such as antibiotics, toxins, and antitoxins, and stress response agents of various nature (1). Being important industrial producers of antibiotics and vitamins (2), as well as the source of plant hormones beneficial to their plant hosts (3), the *B. subtilis* group bacteria provide great incentive to discovering and describing more of their potentially useful strains.

The Can02R strain was isolated from the roots of the resurrection plant host, *Chenopodium album* L., collected near the city of Mikhailovsk (45.156812N, 42.098796E) in Russia according to a previously described protocol (4). Plant roots were disinfected with tap water for 30 s, followed by 70% ethanol for 5 min, then 15% H_2_O_2_ for 10 min, and five times of sterile water for 2 min, then subsequently crushed with a pestle in a mortar under sterile conditions. Aliquots of 100 mL of the resulting plant juices were plated on 1/20 tryptic soy agar plates (TSA, Difco Laboratories, MI, USA). Strain Can02R was grown in Luria-Bertani (LB) medium (Thermo Fisher Scientific, MA, USA) at 30°C overnight. The Wizard Genomic DNA Purification Kit (Promega, USA) was used to extract genomic DNA. The DNA library preparation was done with the KAPA HyperPlus Kit (Roche, Switzerland). Whole-genome sequencing was performed using the NextSeq 1000 platform (Illumina, USA) with the NextSeq 1000/2000 P1 Reagents (300 cycles). A total of 2,400,838 reads were acquired with an average length of 150 bp. The sequence quality was estimated using FastQC v. 0.11.9 (5).

*De novo* genome assembly was performed with SPAdes v.3.13.1 (6), applying the ‘--careful’ option. The draft genome assembly contains 3,965,760 bp in five scaffolds with an average coverage of 148.361× after merging contigs using RagTag v. 2.1.0 (7). We assessed the assembly quality with QUAST v. 5.2.0 (8), resulting to N50 = 3,909,444 and a GC content = 46.61%. The application of Prokka v1.14.5 (9) resulted in the prediction of 3,893 protein-coding sequences and 96 RNA-coding sequences. The National Center for Biotechnology Information (NCBI) prokaryotic annotation pipeline (10) was used as well. We were able to define the species of the isolate as *Bacillus amyloliquefaciens* (98.8519% identity of the genome to the NCBI RefSeq assembly GCF_019396925.1) using FastANI v1.34 (11). Assembly assessment with BUSCO v 5.2.2 (12) revealed 99.8% complete and single-copy BUSCOs and the remaining 0.2% of fragmented BUSCOs in the Bacillales database.

We searched for secondary metabolite-associated gene clusters with the antiSMASH v7.1.0 (13) and KEGG Annotation tools (14). We were able to identify genes encoding the synthesis of several antibiotics, as well as components of antibiotic and multidrug resistance systems. A plipastatin/fengycin gene cluster was found responsible for anti-fungal activity. In Table 1, we provide a more detailed list of notable genes.

**TABLE 1:** Annotated gene products that can be involved in adaptive responses

Anti-fungal activity	Plipastatin synthase
	Fengycin
Antibiotics synthesis and resistance	Gramicidine synthase
	Penicillin-binding complex, PbpX
	Bicyclomicin resistance protein
	Bacitracin transporter
Stress-resistance	Salt-stress responsive protein, YocM
	Stress response protein, NhaX
	Arsenical-pump membrane protein, ArsB
Multidrug resistance	Multidrug resistance ABC transporters, YheI and YheH
Other	Fluoride ion transporter, CrcB
	Polyketide synthase
	Surfactin

## Data Availability

The complete genome sequences and raw reads were deposited in National Center for Biotechnology Information (NCBI) and can be accessed under BioProject PRJNA1155005, SRA SRR30503330, and assembly GCA_041947055.1. Prokka and antiSMASH annotations are available on Figshare (https://doi.org/10.6084/m9.figshare.27301797.v1).
